# Awareness with paralysis and symptoms of post-traumatic stress disorder among mechanically ventilated emergency department survivors (ED-AWARENESS-2 Trial): study protocol for a pragmatic, multicenter, stepped wedge cluster randomized trial

**DOI:** 10.1186/s13063-023-07764-5

**Published:** 2023-11-25

**Authors:** Brian M. Fuller, Brian E. Driver, Michael B. Roberts, Christa A. Schorr, Kathryn Thompson, Brett Faine, Julianne Yeary, Nicholas M. Mohr, Ryan D. Pappal, Robert J. Stephens, Yan Yan, Nicholas J. Johnson, Brian W. Roberts

**Affiliations:** 1 Department of Anesthesiology, Division of Critical Care, Department of Emergency Medicine, Washington University in St. Louis School of Medicine, St. Louis, MO 63110 USA; 2 Department of Emergency Medicine, Hennepin County Medical Center, 701 Park Avenue, Minneapolis, MN 55415 USA; 3 Department of Institutional Research, Department of Psychology, Philadelphia College of Osteopathic Medicine, Rowland Hall, 514B, 4190 City Avenue, Philadelphia, PA 19131 USA; 4 Cooper Research Institute, Cooper University Health Care, One Cooper Plaza, Dorrance, Camden, NJ 08103 USA; 5 Department of Emergency Medicine, University of Washington/Harborview Medical Center, 325 9th Avenue, Seattle, WA 98104 USA; 6 Departments of Emergency Medicine and Pharmacy, Roy J. and Lucille A. Carver College of Medicine, University of Iowa College of Pharmacy, 200 Hawkins Drive, 1008 RCP, Iowa City, IA 52242 USA; 7 Emergency Department, Charles F. Knight Emergency and Trauma Center, Barnes-Jewish Hospital, 1 Barnes Jewish Hospital Plaza, St. Louis, MO 63110 USA; 8 Departments of Emergency Medicine and Anesthesiology, Division of Critical Care, Roy J. and Lucille A. Carver College of Medicine, University of Iowa, 200 Hawkins Drive, 1008 RCP, Iowa City, IA 52242 USA; 9 Department of Emergency Medicine, Beth Israel Deaconess Medical Center, 330 Brookline Avenue, Boston, MA 02215 USA; 10 Department of Medicine, Division of Critical Care Medicine, University of Maryland School of Medicine, 655 W. Baltimore Street, Baltimore, MD 21201 USA; 11 Division of Public Health Sciences, Department of Surgery, Division of Biostatistics, Washington University School of Medicine, 418E, 2Nd Floor, 600 South Taylor Ave., St. Louis, MO 63110 USA; 12 Departments of Emergency Medicine and Medicine, Division of Pulmonary, Critical Care, and Sleep Medicine, University of Washington/Harborview Medical Center, 325 9th Avenue, Seattle, WA 98104 USA; 13 Department of Emergency Medicine, Cooper Medical School of Rowan University, One Cooper Plaza, K152, Camden, NJ 08103 USA

**Keywords:** Emergency medicine, Awareness with paralysis, Sedation, Post-traumatic stress disorder, Mechanical ventilation, Critical care

## Abstract

**Background:**

Awareness with paralysis (AWP) is memory recall during neuromuscular blockade (NMB) and can cause significant psychological harm. Decades of effort and rigorous trials have been conducted to prevent AWP in the operating room, where prevalence is 0.1–0.2%. By contrast, AWP in mechanically ventilated emergency department (ED) patients is common, with estimated prevalence of 3.3–7.4% among survivors given NMB. Longer-acting NMB use is a critical risk for AWP, and we have shown an association between ED rocuronium use and increased AWP prevalence. As NMB are given to more than 90% of ED patients during tracheal intubation, this trial provides a platform to test an intervention aimed at reducing AWP. The overall objective is to test the hypothesis that limiting ED rocuronium exposure will significantly reduce the proportion of patients experiencing AWP.

**Methods:**

This is a pragmatic, stepped wedge cluster randomized trial conducted in five academic EDs, and will enroll 3090 patients. Per the design, all sites begin in a control phase, under observational conditions. At 6-month intervals, sites sequentially enter a 2-month transition phase, during which we will implement the multifaceted intervention, which will rely on use of nudges and defaults to change clinician decisions regarding ED NMB use. During the intervention phase, succinylcholine will be the default NMB over rocuronium. The primary outcome is AWP, assessed with the modified Brice questionnaire, adjudicated by three independent, blinded experts. The secondary outcome is the proportion of patients developing clinically significant symptoms of post-traumatic stress disorder at 30 and 180 days after hospital discharge. We will also assess for symptoms of depression and anxiety, and health-related quality of life. A generalized linear model, adjusted for time and cluster interactions, will be used to compare AWP in control versus intervention phases, analyzed by intention-to-treat.

**Discussion:**

The ED-AWARENESS-2 Trial will be the first ED-based trial aimed at preventing AWP, a critical threat to patient safety. Results could shape clinical use of NMB in the ED and prevent more than 10,000 annual cases of AWP related to ED care.

**Trial registration:**

ClinicalTrials.gov identifier NCT05534243. Registered 06, September 2022.

**Supplementary Information:**

The online version contains supplementary material available at 10.1186/s13063-023-07764-5.

## Background

Awareness with paralysis (AWP) is explicit memory recall during neuromuscular blockade (NMB) [1–3]. It is crippling and can cause catastrophic psychological harm including post-traumatic stress disorder (PTSD), depression, anxiety, complex phobias, and lead to suicide [3–9]. Decades of rigorous investigations on hundreds of thousands of patients have been completed with the goal to prevent AWP in the operating room, where prevalence is approximately 0.1–0.2% [3, 10].

**Fig. 1:**
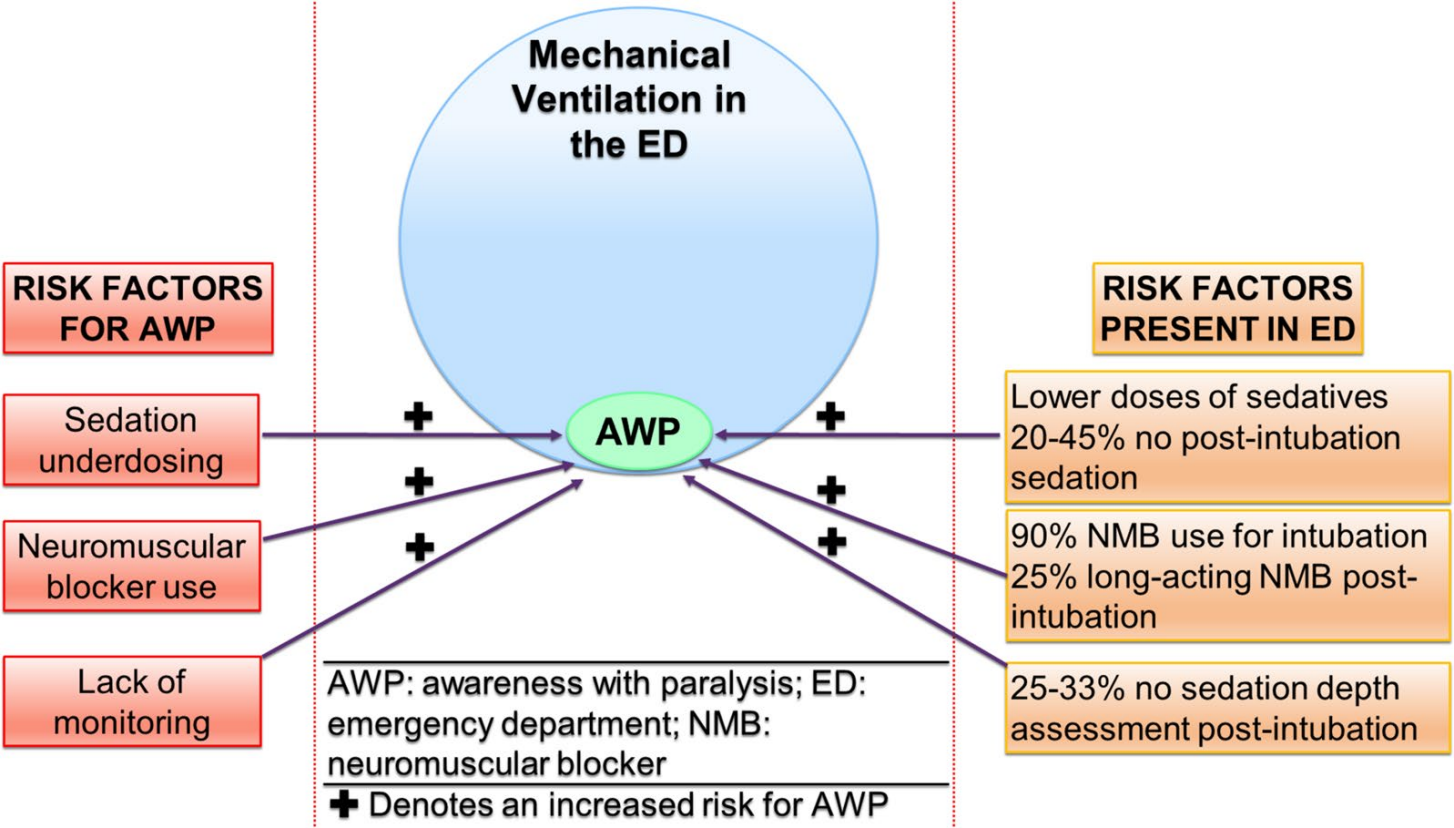
Important risk factors for awareness with paralysis include sedation under dosing, neuromuscular blocker use, and a lack of protocolled monitoring of sedation depth. Every major risk factor for awareness with paralysis commonly occurs in the emergency department

By contrast, comparatively little investigation into AWP has occurred among emergency department (ED) patients, where mechanical ventilation is required for almost 400,000 patients annually in the USA [11]. Important risk factors for AWP identified in operating room patients include (1) use of longer-acting NMB; (2) lack of sedation depth monitoring; (3) total intravenous (versus inhaled) anesthesia; and (4) under dosing of anesthesia [1–3, 10]. Clinical data suggests that ED sedation practices place patients at high risk for AWP (Fig. 1). These practices include a lack of any analgesia and sedation for 20–45% of ED patients after intubation, with up to 33% of patients with no sedation depth assessment while mechanically ventilated in the ED [12, 13]. In addition, approximately 90% of patients are given NMB to facilitate tracheal intubation in the ED, with an increasing use of longer-acting NMB such as rocuronium (versus succinylcholine) [14]. Since these patients cannot move, nor relay pain or fear, they commonly receive lower doses of sedation, and in delayed fashion, with up to 25% receiving sedation after a delay of ≥ 50 min [13–16]. Importantly, high variability exists between sites, as the ED-SED Study demonstrated a wide range of (1) no sedation (site ranges, 5.9–52.6%); (2) post-intubation NMB use (0–27.3%); and (3) sedation monitoring (0–100%) [17]. Similarly, in a secondary analysis of the LOTUS-FRUIT study, 26% of ED patients were given no sedation, and 70% had no sedation depth assessed (site ranges, 0–100%) [18].

The data cited above provided our research group with the preliminary data to justify further study of AWP among mechanically ventilated ED patients. The ED-AWARENESS Study was a single-center, prospective cohort study which consecutively enrolled 383 mechanically ventilated ED patients over a 12-month period at a large academic medical center [19, 20]. Among survivors given NMB in the ED, the proportion of patients experiencing AWP was 3.3% (10/306; 95% CI, 1.6–5.9%) [19]. In an a priori secondary analysis of the multicenter ED-SED Pilot Trial, we demonstrated that 4.1% (13/314; 95% CI, 2.2–7.0%) of survivors that were given a NMB in the ED experienced AWP [21–23]. Finally, in a single-center prospective cohort conducted at Hennepin County Medical Center (an enrolling site for the current trial), the proportion of patients experiencing an AWP event was 7.4% (66/886; 95% CI, 5.8–9.4%) [24]. Taken as a whole, these data justify a clinical trial aimed at prevention of AWP among mechanically ventilated ED patients and strongly suggest the ED could be a high-yield arena in which to improve patient-centered clinical outcomes.

Psychological trauma and PTSD are a significant burden among the critically ill. PTSD is the development of significant distress or social impairment in someone directly or indirectly exposed to death, threated death, injury, or violence. Critical illness is psychologically traumatic, and 25–35% of survivors experience PTSD [25–27]. These survivors have worse quality of life, greater frequency of pain and substance abuse, increased healthcare cost, and threefold greater risk of death [28–33].

**Fig. 2:**
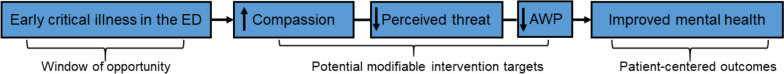
Example novel paradigm and hypothesized causal pathway for improving patient-centered outcomes. It is possible that by addressing the early period of critical illness in the emergency department, modifiable targets (increasing provider compassion, decreased patient-perceived threat, preventing awareness with paralysis) can be used to improve patient-centered clinical outcomes

However, similar to AWP, there is a significant knowledge gap with respect to critically ill, mechanically ventilated ED patients. Prior ED-based PTSD work has largely focused on traumatic injury or specifically excluded mechanically ventilated patients [34–37]. In a cohort study of 99 ED patients, our team demonstrated clinically significant PTSD symptoms in 31% of patients with acute respiratory failure, but only 9 patients (9.1%) were mechanically ventilated [38]. Therefore, the burden of PTSD in this cohort is unknown, and the ED could be an optimal target for improving mental health outcomes for several reasons. Critical illness-related mental health disorders are linked to exposures during care, and early frightful memories during illness are linked to higher PTSD prevalence [1, 26]. In addition, delays in care for PTSD are common and linked to refractory symptoms [6, 39–41]. This evidence suggests the early period of critical illness opens a window to reduce psychological morbidity in survivors. The ED-AWARNESS-2 Trial will inform creation of interventions to be used during (not after) an event to mitigate symptom development, and it will use the ED as a target in which to improve outcomes during the “golden hours” (Fig. 2) [34, 42].

There is significant rationale to target rocuronium use in the ED as a means to prevent AWP. In the ED-AWARENESS Study and ED-SED Pilot Trial, 19/23 (92.6%) AWP patients received rocuronium [19, 23]. In multivariable analysis, rocuronium was independently associated with AWP (adjusted odds ratio, 6.05; 95% CI, 1.46–17.56) [19, 23]. Importantly, AWP was not associated with other sedation-related variables, including (1) sedation monitoring; (2) analgesics, sedatives; or (3) sedation depth. This confirms that a proposal to target ED-based rocuronium may have the greatest chance to improve safety and prevent AWP. This is critical as data from the National Emergency Airway Registry (*n* = 17,583) show rocuronium use was 5% for intubations in 2002, yet up to ~ 45% by 2012 [14]. Use was up to 49% in our ED SED Study, and 60% in the ED-SED Pilot Trial [17, 21]. This indicates that AWP is likely a growing health problem. Contrary to a 5-min duration of succinylcholine (the historical default NMB), rocuronium can paralyze for up to 150 min, greatly increasing AWP risk. In addition, in clinical trials comparing rocuronium versus succinylcholine, with respect to intubating conditions, data has either favored succinylcholine or shown no difference in outcomes [43–45]. However, other preliminary work from our research team did not show an association between rocuronium and AWP risk, but rather pre-intubation mental status [24]. This suggests equipoise in the data and further confirms the need for this large-scale clinical trial.

Our objective is to conduct a pragmatic clinical trial aimed at limiting rocuronium exposure in order to reduce the proportion of mechanically ventilated ED patients experiencing AWP. We hypothesize that limiting rocuronium exposure in the ED, using succinylcholine as the default NMB of choice, will significantly reduce the proportion of patients experiencing AWP.

## Methods

### Trial design

**Fig. 3:**
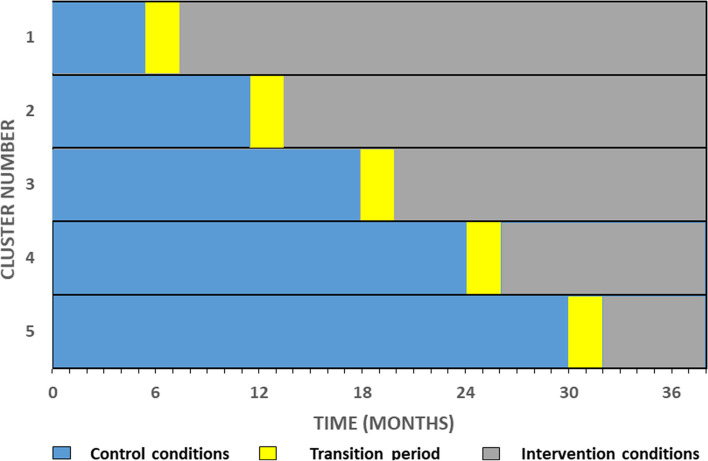
Stepped wedge cluster randomized trial where clusters cross to intervention every 6 months (step duration), with a preceding 2-month transition to implement the intervention

This is a multicenter, pragmatic, stepped wedge cluster randomized superiority trial enrolling at five academic sites over an approximately 3.5-year period, divided into six time periods of 6 months each. A schematic of the trial design appears in Fig. 3. Prior to the trial, each site (one site equals one cluster) was randomly allocated to their position within the design. One cluster will cross to the intervention period, at which point succinylcholine will become the default NMB in the ED, every 6 months (i.e., step duration) from the 2nd to the 6th time period. Cluster order will be determined by computer-based randomization, conducted and generated by the trial statistician (YY), and concealed from each site. To begin, each site will be exposed to control conditions; by the end of the trial, each site will be exposed to the intervention. Enrollment began in June of 2023 and we anticipate recruitment of the total sample size to be completed by September of 2026.

A stepped wedge cluster randomized design was chosen for several reasons. It is a robust approach to test quality improvement interventions, such as that in the ED-AWARENESS-2 Trial, and is a rigorous way to study interventions during routine implementation [46, 47]. In addition, randomization of individual patients would be logistically infeasible and yield a small and very selective study population, undermining external validity. Stepped wedge designs are naturalistic because implementation can proceed much as it would without the trial and are also rigorous because they provide randomized evidence of effectiveness [47]. The design also allows between- and within-group comparisons, enhancing statistical power [47–49]. Finally, a stepped wedge study is ideal when evidence already exists, yet there is suboptimal uptake; based on years of operating room research and our preliminary data, we know an effective way to prevent AWP is to limit use of longer-acting NMB, such as rocuronium [3, 19, 23]. The increase in rocuronium use in the ED over the past several years has occurred without any robust randomized evaluation of patient-centered outcomes, as outcomes have typically centered around intubation success rate [14]. The unintended consequence is an incidence of AWP that threatens patient safety. The stepped wedge design provides a means to conduct the first patient-centered, randomized evaluation on this topic and optimizes enrollment due to potential benefits of the intervention while the trial is ongoing.

### Study setting and population

This trial will target mechanically ventilated adult patients in the ED of five academic medical centers. Inclusion criteria are (1) mechanical ventilation via an endotracheal tube; (2) age ≥ 18 years; and (3) treatment with a NMB in the ED (for tracheal intubation or in the post-intubation phase of care). Exclusion criteria are (1) acute or chronic neurologic injury with deficit that prevents assessment of AWP (i.e., stroke, intracranial hemorrhage, traumatic brain injury, cardiac arrest, advanced dementia); (2) death before extubation; and (3) transfer to another hospital from the ED.

Patients will be recruited from the ED at five academic medical centers in the USA: (1) Cooper Medical School of Rowan University, Camden, NJ; (2) University of Iowa, Iowa City, IA; (3) Hennepin County Medical Center, Minneapolis, MN; (4) Barnes-Jewish Hospital/Washington University School of Medicine, St. Louis MO; and (5) Harborview Medical Center, Seattle, WA. Washington University will serve as the Clinical Coordinating Center (CCC) for the trial. The CCC is composed of the overall trial PI, research coordinators, and grants management team, and is responsible for the administration and management of the trial. Mechanically ventilated patients in the ED reflect the composition of the demographics at each of the sites in this study. Based on our preliminary data, we project that the enrollment of women will be approximately 45% and the enrollment of minorities will be approximately 35% Black or African-American, 10% Asian, and 10% Hispanic. We will not exclude any subjects based on gender, race, or ethnicity. We therefore expect that the study findings will hold external validity.

### Screening and study initiation

This study will identify those patients who present to the ED and require mechanical ventilation. Each site has a system in place for real-time alerts (24 h/day) when mechanical ventilation is used in the ED, and has validated its notification system from the ED to ensure the population of potentially eligible patients will be consecutive mechanically ventilated patients presenting to the ED. All patients that satisfy inclusion and exclusion criteria will be enrolled.

### Interventions

Patients in the control phase of the trial will receive usual, clinician-directed care. To limit a Hawthorne-like effect, clinicians will have no knowledge of the study during the control. After 6 months, as determined by computer-generated randomization, a site will enter a 2-month transition phase, during which we will implement a multifaceted intervention aimed at reducing the use of rocuronium in the ED and consequently increase the use of succinylcholine. The intervention will be delivered at the cluster level, and the strategies employed revolve around the use of “nudges” without restricting clinician choice [50]. Nudges can be a powerful strategy to modify clinician behavior in order to increase uptake of evidence-based practices [48, 50–52]. A type of nudge involves the use of “defaults”; default options are those set in place when no alternatives are actively chosen and they have been shown to positively influence clinician decisions [50]. Succinylcholine will be the default NMB over rocuronium because (1) it has been the default NMB of choice in the ED for > 40 years [53]; (2) its 5-min duration of action greatly reduces AWP risk; (3) our preliminary data regarding AWP and its association with rocuronium [19, 23]; (4) ED rocuronium use has increased despite no patient-centered studies showing benefit over succinylcholine [17, 21]; and (5) a robust body of literature which documents equivalent efficacy and safety profiles of succinylcholine and rocuronium [54–56].

A basic challenge of pragmatic clinical trials is to design an intervention that is appropriately balanced with respect to flexibility (i.e., it must be adaptable to local culture) and adherence (i.e., it must cause some level of change in order for efficacy to be adequately tested). In addition, a fundamental aspect of nudges is their influence on decisions without restricting choice. Due to these issues, our multifaceted intervention strategy will have both mandatory (standardized) and optional interventions during the transition phase. For the mandatory interventions, we will educate and engage (1) with emergency medicine (EM) physician and nursing leadership to secure endorsement of the trial; and (2) to deliver standardized presentations and lectures to clinicians and trainees (e.g., grand rounds, faculty meetings). We will also use passive alerts in the form of (1) strategically placed laminated graphics which advertise and promote the trial in the ED; and (2) distribution of a one-page document, which contains the background, rationale, and goals of the trial in one convenient location. Finally, active alerts will include (1) announcement of succinylcholine as the default NMB in the nursing “huddle” (before shifts); and (2) monthly emails to clinicians showing the breakdown of NMB use (continuous quality improvement). Optional interventions will be dictated by local culture and resources, or as needed if intervention fidelity declines over time. These include the use of ED-based pharmacy resources to advertise and promote the trial, as well as modification of access to rocuronium, making succinylcholine a more “convenient” option to clinicians (e.g., in the bedside airway box versus the automated medication dispensing system). If needed, we will identify more local champions, in addition to each site principal investigator, to participate in and promote the trial. Finally, if needed we will send emails to individual clinicians each time rocuronium is used in order to explore the rationale for its choice. This provides *accountable justification* regarding NMB choice (i.e., justification of the decision to not use the default). After the 2-month transition phase, each site will begin the intervention phase, in which enrollment will be resumed.

We will aim to prevent drift of intervention fidelity over time in several ways. Sites with an earlier transition to the intervention will be able to advise subsequent sites on troubleshooting, potential pitfalls, and successes. In addition, the active alerts (see above) will provide routine reminders to the clinical staff regarding the trial. We will also monitor the breakdown of NMB use on a rolling and near-continuous basis and present these results at our ED-AWARENESS-2 team meetings. If problems with treatment fidelity are observed, we will re-educate clinical staff at the site and tailor the intervention re-education as needed. In addition, we will more uniformly employ the optional interventions in order to increase intervention fidelity. In this way, we not only have uniform and mandatory interventions to be employed at the onset of the trial at each site, but also maintain optional interventions which can be strategically “in our back pocket” if needed.

### Informed consent

We will conduct the study with waiver of the requirement to obtain signed informed consent. The control phase is entirely observational. The intervention uses nudges and defaults for quality improvement, according to guidelines and recommendations regarding AWP prevention. In addition, for continued monitoring and quality improvement, the assessment of AWP is part of standard routine post-intubation care for millions of mechanically ventilated patients undergoing anesthesia annually in the USA, and it is also part of ongoing quality improvement initiatives in mechanically ventilated ED patients. However, all care will remain at the discretion of treating clinicians, and data during the intervention phase will also be collected under observational conditions. Specifically, while our intervention is aimed at reducing the use of rocuronium in the ED in order to prevent AWP, the final choice of all treatment decisions will remain at the discretion of the treating clinical team at all times. The only processes for research are administration of the mental health and quality of life questionnaires, at days 30 and 180, and medical record review. Verbal informed consent will be obtained prior to administration of the day-30 and day-180 follow-up questionnaires.

Washington University in St. Louis will act as the single institutional review board for this trial. The study protocol has received ethical approval (Additional File 1) by the Human Research Protection Office at Washington University School of Medicine in St. Louis (IRB # 202207132).

### Outcome measures

The primary outcome is the proportion of patients experiencing AWP in the control phase versus the intervention phase. Our methodology to assess for AWP is congruent with large operating room trials, and our prior approach [19, 20, 57, 58]. The modified Brice questionnaire has been used for > 50 years to assess for AWP and will be used in this trial (Additional File 2) [59]. To be considered for AWP, patients must report memory of paralysis either before (i.e., recall of intubation) or after (i.e., waking with paralysis) losing consciousness [19]. Because of simplicity and excellent diagnostic accuracy, the Richmond Agitation-Sedation Scale (RASS) will be used to screen for delirium and before assessment of AWP [60]. AWP will be assessed at one time point. Trained team members who will check the medical record daily for extubation and assess for AWP before hospital discharge, under waiver of the requirement to obtain signed informed consent. In patients discharged during off hours (e.g., weekends, night), we will assess AWP via telephone. The final determination of AWP will be independently adjudicated by three experts who will be provided questionnaire responses, qualitative reports of patient experience, and pertinent clinical data. Reviewers will adjudicate events as no, possible, or definite AWP, and determined when ≥ two agree. If all hold opposing views, a fourth reviewer will assist. Reviewers will be blinded to NMB, sedation medications, and trial phase.

Secondary outcomes include mental health and quality of life assessments at 30 and 180 days after hospital discharge, assessed via telephone follow-up after obtaining verbal informed consent. Questions will be asked in reference to the index hospitalization. PTSD symptoms will be measured using the PTSD Checklist for DSM-5 (PCL-5), a validated 20-item (score 0–4, Likert scale) self-reported measure that assesses 20 DSM-5 symptoms of PTSD (clinically significant symptoms = PCL-5 > 32) [61]. While PTSD is our main secondary outcome of concern, depression, anxiety, and impaired quality of life (QOL): (1) often co-exist with PTSD; (2) are common after critical illness; and (3) are also sequela linked to AWP [1, 2, 5, 7, 10, 25, 62, 63]. To give a broad picture of burden endured by survivors, we will assess for depression and anxiety with the Hospital Anxiety and Depression Scale (HADS), a validated 14-item (7 depression, 7 anxiety, ordinal score 0–3) questionnaire commonly used in critical illness [25]. We will evaluate QOL with EuroQol-5D (EQ-5D), the most widely used scale for measuring health-related QOL and part of the core sets recommended for critical care studies on post-discharge outcomes [64, 65].

To assess for potentially modifiable intervention targets for future trials aimed at PTSD symptom prevention, we will collect patient perception of (1) perceived threat; (2) clinician compassion; and (3) degree of family/friend emotional support. Perceived threat will be measured with the validated 7-item tool used in our prior work [19, 38, 66, 67]. Patient perception of clinician compassion will be assessed with a 5-item measure, developed, and validated by our team (only instrument validated for ED use) [68]. Family/friend emotional support during hospitalization will be assessed with two items previously used in the ED as measures of anxiety-provoking social support that are significantly associated with PTSD symptoms: (1) “How much of the time did your support person need you to comfort them?”; and (2) “How much of the time did your support person make you anxious?” [69].

### Data

Patient-level data will be easily accessible from the electronic medical record. The following baseline characteristics will be collected: age, gender, race, weight, height, pre-existing comorbid conditions, vital signs at presentation, and pertinent laboratory variables. Pre-intubation mental status, drugs used to facilitate intubation, and ventilator settings in the ED will be collected. After the initiation of mechanical ventilation in the ED, all medications related to analgesia and sedation in the ED will be collected. Sedation depth in the ED will be recorded by bedside nurses, using validated sedation depth scales, such as RASS or the sedation-agitation scale (SAS), per existing protocols [70].

The following in-hospital data will be collected: duration of ventilation, agents used for analgesia and sedation during the first 24 h of ICU admission, depth of sedation during the first 7 days after admission, lengths of stay in the ICU and hospital, discharge location, and mortality status (Table 1).

**Table 1:** Schedule of screening, data collection, and outcomes assessments for the ED-AWARENESS-2 Trial

Measurement/event	ED	Extubation	^a^1	2	3	4	5	6	7	MV^b^	HOSP D/C	Day 30	Day 180
Inclusion criteria	X												
Exclusion criteria		X											
Demographics, baseline data	X												
Laboratory values	X												
Blood gas values	X		X										
ED procedures and care	X												
Intubation meds and location	X												
Safety events (i.e., intubation success, unplanned extubation, cardiac arrhythmia, allergy, MH)	X												
Ventilator settings/data	X												
Sedation depth (RASS, SAS), documented by bedside nurse	X		X	X	X	X	X	X	X				
Medications for sedation, analgesia, NMB	X		X										
Delirium assessment/CAM-ICU			X	X	X	X	X	X	X				
Assessment of AWP		X											
Ventilator duration										X	X		
Length of stay (hospital and ICU)											X		
Discharge location											X		
Mortality status											X		
Mental health and QOL assessments												X	X

*ED* emergency department; *MV* mechanical ventilation; *MH* malignant hyperthermia; *RASS* Richmond Agitation-Sedation Scale; *SAS* Sedation-Agitation Scale; *NMB* neuromuscular blockade; *CAM* confusion assessment method; *ICU* intensive care unit; *QOL* quality of life

^a^The numbers 1–7 refer to hospital days, with hospital day 1 referring to the first day after admission from the ED

^b^If patient is extubated for 24 h or more, then that is the end of ventilation duration. If patient is extubated and re-intubated within 24 h, this is treated as though they were never extubated

### Data management and quality control

Data will be managed using Research Electronic Data Capture (REDCap), including regulatory document management, data entry and validation, source document and progress monitoring, subject tracking, and secure data transfer [71, 72]. During study initiation, weekly communication will occur with each site via video conference. During these initial meetings, REDCap data fields and entry will be reviewed in detail, tested at each site, and troubleshooting and modification will occur as needed to streamline efficient data entry. Throughout the study, data checks for valid ranges and completeness of data will occur. We will track and identify for each subject the study forms that are expected but not yet entered into REDCap. All site personnel will have ability to run reports that will provide detailed lists of expected forms and of all data queries for their site. In addition, the CCC will provide reports to site investigators, as needed, for timely submission of data forms and resolution of queries. All data will be entered into REDCap by site personnel.

Excluding questionnaires, all data will be obtained from the medical record and verified before data entry. In REDCap, validation rules will ensure acceptable ranges, and valid and accurate data, which will be monitored for errors on an ongoing basis. Any concerns or discrepancies will be resolved by study sites and the CCC; re-training will occur as needed. Routine monthly and ad hoc reports will be run as part of quality control. The reports will track patient accrual and status, data completeness, and responses to data queries.

Data entered into REDCap will be stored on Washington University servers. User privileges related to data management, beyond data entry, will be restricted. As standard operating procedure, use of REDCap to enter and store data, in a secure and password-protected manner, provides certainty that data will not be lost. After study completion, de-identified data will be exported into a statistical analysis package and analyses will proceed in a way such that patient confidentiality is maintained.

### Safety monitoring

A data and safety monitoring board (DSMB), independent of the research team and sponsor, will be used to monitor the data. The DSMB will assess safety and efficacy of trial procedures and monitor the overall conduct of the trial. The DSMB also will review adverse event data, other safety data, quality and completeness of data, protocol adherence data, and enrollment data at each meeting to ensure proper trial conduct and continued feasibility of answering the research questions. Meetings between the research team and the DSMB will occur before the study begins, after 25% of the total sample size is accrued each time, and upon trial completion.

The intervention is designed to enhance patient safety by quality improvement related to the selection of neuromuscular blockers by clinicians. Consequently, we aim to see an increase in succinylcholine use during the intervention phase of the trial, which is congruent to clinical practice for more than 40 years and consistent with recommendations for preventing awareness with paralysis. There is over a decade of research on tens of thousands of patients showing equivalent intubation success rates between succinylcholine and rocuronium. In addition, intubation success rate in the ED approaches 100% [14, 54]. We therefore, do not expect to see any difference in intubation success rate between the control and intervention phases, but will track and report this measure. Given the typically small and transient increase in serum potassium of ~ 0.5 to 1 mEq/L known to occur with succinylcholine, there is the potential for an increase in cardiac arrhythmias related to hyperkalemia to occur in the intervention phase. The overall incidence of cardiac dysrhythmia in the peri-intubation period in the ED is approximately 0.7% with no difference between succinylcholine and rocuronium. We therefore do not expect to see any difference in this adverse event between the control and intervention phases, but will track and report this measure [14]. Other very rare adverse events associated with succinylcholine include malignant hyperthermia (incidence ~ 0.009%) and allergic reaction to succinylcholine (incidence 0.01%) [73, 74]. Both of these adverse events are quite unlikely to occur, but will be tracked and reported. Unexpected events will be reported by the site principle investigators to the CCC as detected.

Adverse events and serious adverse events will be presented at each DSMB meeting. Adverse events will be classified according to severity, relatedness, and expectedness. Each site principle investigator will designate their first impression of the seriousness and relatedness of the adverse events, and this will be forwarded to a centralized independent safety monitor, who will adjudicate final relatedness, seriousness, and expectedness. As the control phase is entirely observational, this review process will pertain to sites after their transition to the intervention phase of the trial. Events will be reported in trial publications.

As the adverse events related to the choice of a particular neuromuscular blocker are so rare, and most adverse events will be related to critical illness itself, we have not established formal stopping rules. Recommendations by the DSMB will be made based on the assessment of clinical outcomes, adverse events, and human subjects risk in totality.

### Proposed statistical methods

Patient characteristics will be reported using descriptive statistics and frequency distributions. Continuous variables will be compared using independent samples *t*-test or Mann–Whitney *U* test, and categorical variables will be compared using chi-square test or Fisher’s exact test.

AWP will be calculated as proportion (with 95% confidence intervals [CI]) of patients with possible or definite events, as done before [19, 20, 57]. Adjudicator agreement will be assessed, per prior approach, with two-way, random effects, intraclass correlation coefficient for absolute agreement according to 0 = no awareness, 1 = possible awareness, and 2 = definite awareness. All analyses will be intention to treat, such that all patients will be evaluated, regardless of clinician adherence during the intervention phase. The unit of randomization will be the ED, and the unit of analysis will be the patient. The primary analysis will compare the proportion of patients with AWP in the control phase versus the intervention phase. To estimate the intervention effect, mixed effects models and generalized estimating equations (GEE) are most commonly used in stepped wedge designs. However, due to the smaller number of clusters (*n* = 5), it is more appropriate to use a generalized linear model, with cluster included as a fixed effect (i.e., a logistic regression model for binary outcome data with each of five sites as fixed effect) [75–78]. We will first fit a basic model, in which the intervention effect is estimated, adjusting for secular trends (i.e., time effect). We will then extend the basic model by incorporating and testing appropriate interaction terms to test the nature of the intervention effect. Examples include (1) an intervention-by-cluster interaction for heterogeneity of the intervention effect across clusters; and (2) an intervention-by-time for heterogeneity of the intervention effect across time. A possible delayed intervention effect will be tested by changing the intervention indicator (i.e., 0 or 1) into some fractions between 0 and 1, aligned with the time from switching from control to intervention. Unadjusted analyses will also be reported.

Instrumental variable (IV) methods will be used to account for non-adherence, as there could be differences between intervention effectiveness (i.e., effect among all patients to whom the intervention is targeted) and efficacy (i.e., effects among patients whose clinicians adhere to the intervention). We will use a non-parametric IV estimator adapted to stepped wedge designs, and IV regression methods to estimate complier average causal effect (CACE) of the intervention [79]. To avoid conflating the effect of time with the intervention effect, we will (1) obtain step-specific CACE estimates and (2) summarize step-specific estimates across all steps. We will also use two-stage residual inclusion to perform IV regression, using individual data from all six time points, adjusting for time and cluster effects [80–82]. Unlike per-protocol analyses, CACE analyses are not biased by selection effects and have been shown to be valid inferential approaches in stepped wedge trials [79].

We will determine proportion (with 95% CIs) of patients with clinically significant PTSD symptoms (PCL-5 score > 32) at 30 days and 180 days post-discharge [83, 84] and compare the control versus the intervention phase. To test the independent association of AWP (main exposure of interest) with 30-day PTSD symptom development, multivariable logistic regression will be used, with covariates selected for inclusion a priori [85]. The model will be adjusted for covariates previously associated with PTSD symptoms in critical illness survivors: (1) age; (2) sex; (3) prior psychological trauma (PC-PTSD-5 score)/mental health history; (4) illness severity; and (5) receipt and dose of benzodiazepines [10, 25, 26, 62, 86, 87]. We will adjust for race, given known racial disparities in PTSD symptoms [88–91]. To examine time trends in PTSD symptoms, and independent effects of AWP on time trends, mixed effect logistic regression will be used. Individual random intercept and random time slope will be specified and tested along with other potential risk factors. We will identify individual-level potential risk factors which could explain variation in individual time trend by testing interaction between random time slope and risk factor(s). In all of these models, cluster effect will be modeled as a fixed effect, using cluster indicator in the model. Similar analyses will be conducted for the other secondary outcomes.

**Fig. 4:**
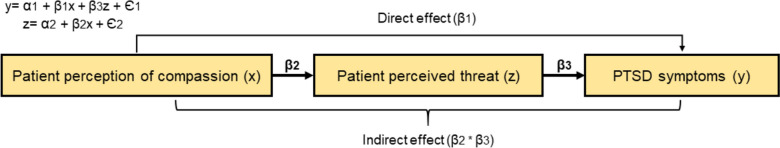
Structural equation model of the direct and indirect effects of perception of compassion on PTSD symptoms

Using structural equation modeling, we will test if patient-perceived threat mediates the relationship (i.e., on the causal pathway) between patient perception of clinician compassion and PTSD symptoms (PCL-5 as a continuous variable) at 30 days. To test for mediation, we will calculate the direct effect of patient perception of compassion on PTSD symptoms, as well as the indirect effect of patient perception of compassion on PTSD symptoms that passes through patient-perceived threat, using bootstrapped standard errors and 95% confidence intervals (Fig. 4) [92, 93]. We will perform separate analyses to also test associations between family/friend emotional support [adjusting for support persons physically present (yes/no)] with perceived threat and PTSD symptoms.

### Sample size justification

Our sample size is designed to detect a difference in the proportion of patients experiencing AWP in the control versus the intervention phase. The sample size must be inflated by estimating the design effect, to account for the intraclass correlation coefficient (ICC). More common outcomes are associated with higher design effects, whereas lower prevalence outcomes, such as AWP, have smaller ICCs [94]. Based on our data, and extensive supporting analyses from more than 100 ICC values obtained from research studies and population health outcomes analyses (*n* > 1,000,000 patients) which show a strong linear association between the ICC and outcome prevalence in clustered binary data, we conservatively estimate an ICC of 0.001 [94]. From our preliminary multicenter data, due to a reduction in rocuronium use, it is reasonable to estimate a reduction in the proportion of patients experiencing AWP from 4.0% in the control phase to 0.2% in the intervention phase (data from the operating room), which would require a total sample size of 1650 patients [95]. To err on the side of conservative assumptions, and to account for possible non-adherence during the study, we will assume a prevalence of AWP of 3.3% in the control phase and 0.6% in the intervention phase. These conservative estimates are based on our prior work (control phase), and the intervention phase event rate is similar to that seen in operating room patients managed with total intravenous anesthesia and when longer-acting NMB is limited [10, 19, 96]. Thus, these calculations are both clinically important (would prevent ~ 10,000 AWP cases annually in the USA) and plausible from supporting preliminary work. Considering there are 5 sites and 6 time periods, for 80% power with *α* of 0.05, we will need a total sample size 3090 patients.

With respect to PTSD, if we estimate that PTSD occurs in 45% of those with AWP [5, 6, 9] and 20% of the rest of the cohort [25–27], assuming *α* = 0.05 and power = 0.80, using two-sided *Z*-test with pooled variance, a sample size of 1192 patients will be required for the multivariable model above. With respect to perceived threat, even if correlation between perceived threat and PTSD symptoms is smaller than our prior work (0.3) then a minimum of 412 patients is estimated to detect the mediated effect of perceived threat between clinician compassion and PTSD symptoms [38, 97, 98].

### Recruitment

The first patient was recruited on June 29, 2023. Our timeline is based on estimates and prior work conducted previously at our enrolling sites [17–19, 21, 99–101]. Between 750 and 1500 patients need mechanical ventilation in the ED at our sites annually. If we conservatively estimate enrollment of 0.5 patients per day at each site at a minimum, with five sites, enrollment will take ~ 3.3 years.

### Dissemination and data sharing

**Fig. 5:**
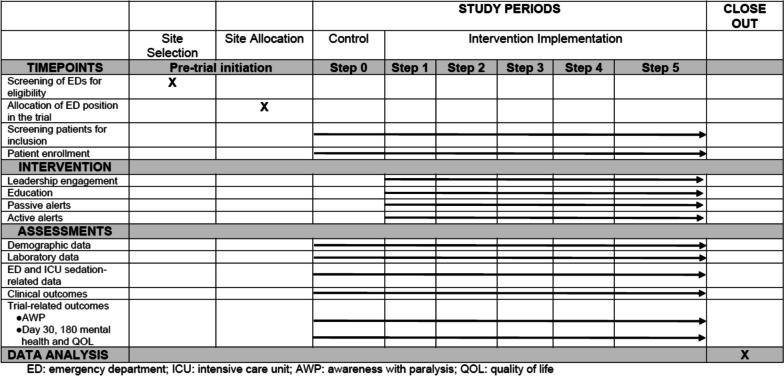
SPIRIT flow diagram for the ED-AWARENESS-2 Trial, a stepped wedge cluster randomized trial

The ED-AWARENESS-2 Trial is registered on ClinicalTrials.gov (NCT05534243). We will follow current data sharing policies of the funding agency in order to share data with other investigators through academically established means. We designed this study in close adherence to the Pragmatic-Explanatory Continuum Indicator Summary-2 (PRECIS-2) criteria for pragmatic trials, and the Consolidated Standards of Reporting Trials (CONSORT) extension for stepped wedge cluster randomized trials [102, 103]. This protocol manuscript also adheres to the SPIRIT reporting guidelines (Additional File 3 (SPIRIT Checklist) and Fig. 5 (SPIRIT Figure)). The protocol and final results will be published in peer-reviewed journals. Data will be presented at annual critical care and EM conferences. At the current time, there are no formal plans for additional studies using these data.

## Discussion

The overall objective of this proposal is to conduct a pragmatic, stepped wedge cluster randomized trial in five academic EDs and to further understand to what extent NMB selection is associated with AWP, our primary outcome. The central hypothesis is that by using nudges and defaults aimed at reducing ED rocuronium use, the proportion of patients experiencing AWP will be significantly reduced. In addition, we hypothesize that the psychological burden suffered by mechanically ventilated ED patients is high, and modifiable targets can be identified. The scientific literature and our preliminary data provide the rationale for conducting this study, and through completion of its aims, we hope to prevent an important threat to patient safety, and develop interventions to be tested in future trials in effort to improve mental health outcomes in survivors going forward. The public health impact from this proposal resides in the fact that it can be readily implemented broadly to prevent thousands of cases of AWP annually. In addition, by targeting the ED and examining psychological outcomes through a patient-centered lens, our results are expected to have a positive public health impact by elucidating the principal pathways of long-term psychological sequelae of critical illness and clarifying the role of time-sensitive ED interventions in contributing to those outcomes. This will allow us to develop specific, targeted countermeasures to improve long-term outcomes for critical illness survivors and identify promising prevention strategies for ED implementation.

This trial has vital strengths. The design and pragmatic nature reflect real-world settings, generating data: (1) with high external validity; (2) with immediate potential for impact; and (3) that are easily disseminated. The analyses are robust and the outcomes (1) are important to patients, clinicians, policy makers, and society; and (2) provide invaluable patient-centered data in patients with acute respiratory failure. As such, findings could change practice by providing: (1) important patient-centered outcome data regarding ED-based NMB use and (2) evidence to target the ED with patient-centered interventions to mitigate mental health morbidity and inequities going forward. The trial will also enroll the largest critically ill cohort to date for the study of AWP.

Relevant limitations exist. A fundamental challenge of pragmatic trials is designing a flexible yet effective intervention-adherence plan to enhance fidelity. To address this, we will employ a systematic and multifaceted intervention approach, similar to our prior pragmatic work that has been successful in ED-based studies. We will perform secondary efficacy (CACE) analyses among patients who would have received the intervention if assigned to it. The modest frequency of AWP may raise questions regarding the number of patients who may benefit. However, as AWP is so consequential to patients experiencing it, our results are expected to carry significant impact. Further, the entire cohort is at high risk to experience psychological morbidity. By rigorously assessing these outcomes in all patients, we will acquire new knowledge that could benefit all mechanically ventilated patients. The trial design includes follow-up beyond the index hospitalization, and incomplete follow-up is a potential problem. We acknowledge loss to follow-up rates ranging from 16 to 50% in prior critical illness-associated PTSD literature [62, 104]. With a response rate of approximately 40%, the sample size will still be large enough to achieve our aims. We will promote patient retention by making the patients aware of the follow-up calls at day 30, obtaining multiple contact numbers by which to reach them, and by messaging the patients to arrange the most appropriate day and time to call.

## Trial status

The current protocol is version 1.0 dated August 9, 2022. The trial initiated enrollment on June 29, 2023, and is expected to be completed in September of 2026.

### Supplementary Information

**Additional file 1. **

**Additional file 2. **Awareness with Paralysis (AWP) Questionnaire.

**Additional file 3. **SPIRIT Checklist for *Trials.*

**Additional file 4. **World Health Organization Trial Registration Data Set items.

## Data Availability

We will follow current data sharing policies of the funding agency (National Heart, Lung and Blood Institute) in order to share data with other investigators through academically established means.

## Abbreviations

AWP: Awareness with paralysis
CACE: Complier average causal effect
CCC: Clinical coordinating center
CI: Confidence interval
CONSORT: Consolidated standards of reporting trials
DSM: Diagnostic and Statistical Manual of Mental Disorders
DSMB: Data and safety monitoring board
ED: Emergency department
EM: Emergency medicine
GEE: Generalized estimating equations
HADS: Hospital anxiety and depression scale
IA: Iowa
ICC: Intraclass correlation coefficient
ICU: Intensive care unit
IRB: Institutional Review Board
IV: Instrumental variable
MN: Minnesota
MO: Missouri
NJ: New Jersey
NMB: Neuromuscular blockade
PC: Primary care
PCL-5: PTSD checklist for DSM-5
PRECIS: Pragmatic-explanatory continuum indicator summary
PTSD: Post-traumatic stress disorder
QOL: Quality of life
RASS: Richmond Agitation-Sedation Scale
REDCap: Research Electronic Data Capture
SAS: Sedation-Agitation Scale
SED: Sedation
WA: Washington
