# Supplementary material for: Awareness with paralysis and symptoms of post-traumatic stress disorder among mechanically ventilated emergency department survivors (ED-AWARENESS-2 Trial): study protocol for a pragmatic, multicenter, stepped wedge cluster randomized trial
Source: Trials. 2023 Nov 25;24:753. doi: 10.1186/s13063-023-07764-5 (PMC10675941; doi:10.1186/s13063-023-07764-5)
Supplement: Supplementary file 1 — Additional file 1. [file 13063_2023_7764_MOESM1_ESM.rtf]

  

Human Research Protection Office			
		
660 South Euclid Ave., Campus Box 8089, St. Louis, MO 63110 Phone: (314) 747-6800

IRB ID #:	202207132

To:		Brian Fuller

Site: 		Washington University in St. Louis

From:		The Washington University in St. Louis Institutional Review Board

Re:	Awareness with Paralysis and Post-Traumatic Stress Disorder among Mechanically Ventilated Emergency Department Survivors: The ED-AWARENESS-2 Trial


Approval Date:	08/17/22
Next IRB Approval
Due Before:	08/16/23

2018 Common Rule/Equivalent Protections Yes

Type of Application:	Type of Application Review:	Approved for Populations:	
 New Project	 Full Board:	 Children	
 Continuing Review	Meeting Date: 08/17/22	     Signature from one parent	
 Modification	 Expedited	     Signature from two parents	
 Modification to add New Site	 Exempt	 Prisoners	
	 Facilitated	 Pregnant Women, Fetuses, Neonates	
		 Wards of State	
		 Decisionally Impaired	

				


					   			
Source of Support:	
	NIH, National Heart, Lung and Blood Institute (NHLBI)
		Awareness with Paralysis and Post-Traumatic Stress Disorder among Mechanically Ventilated Emergency Department Survivors: The ED-AWARENESS-2 Trial (Brian M. Fuller, MD, MSCI)

MATERIALS APPROVED

Protocol:
Protocol Version:  		1.0
Protocol Date:     		08/09/2022

 


Consent/Assent Materials:
	Consent & Assent Forms
		ED-AWARE II consent information sheet-8.1.22.rtf


Questionnaires:
	Subject Data Collection Instruments
		Assessment of symptoms of depression and anxiety.docx
		Assessment of perceived threat.docx
		Assessment of awareness with paralysis.docx
		Assessment of HR QOL.docx
		Assessment of compassion.docx
		Assessment of emotional support.docx
		Assessment of symptoms of PTSD.docx


This approval has been electronically signed by IRB Chair or Chair Designee:
Stephanie Ellerbe, BS, CIP, CCRP
08/19/22 1528
IRB Approval:  IRB approval indicates that this project meets the regulatory requirements for the protection of human subjects.  IRB approval does not absolve the principal investigator from complying with other institutional, collegiate, or departmental policies or procedures.

Recruitment/Consent:  Your IRB application has been approved for recruitment of subjects not to exceed the number indicated on your application form.  If you are using written informed consent, the IRB-approved and stamped Informed Consent Document(s) are available in myIRB.  The original signed Informed Consent Document should be placed in your research files.  A copy of the Informed Consent Document should be given to the subject.  (A copy of the signed Informed Consent Document should be given to the subject if your Consent contains a HIPAA authorization section.)  

Continuing Review:  Federal regulations require that the IRB re-approve research projects at intervals appropriate to the degree of risk, but no less than once per year.  This process is called “continuing review.”  Continuing review for non-exempt research is required to occur as long as the research remains active for long-term follow-up of research subjects, even when the research is permanently closed to enrollment of new subjects and all subjects have completed all research-related interventions and to occur when the remaining research activities are limited to collection of private identifiable information. Your project “expires” at midnight on the date indicated on the preceding page (“Next IRB Approval Due on or Before”).  You must obtain your next IRB approval of this project by that expiration date.  You are responsible for submitting a Continuing Review application in sufficient time for approval before the expiration date, however you will receive reminder notice prior to the expiration date.

Modifications:  Any change in this research project or materials must be submitted on a Modification application to the IRB for prior review and approval, except when a change is necessary to eliminate apparent immediate hazards to subjects.  The investigator is required to promptly notify the IRB of any changes made without IRB approval to eliminate apparent immediate hazards to subjects using the Modification/Update Form. Modifications requiring the prior review and approval of the IRB include but are not limited to:  changing the protocol or study procedures, changing investigators or funding sources, changing the Informed Consent Document, increasing the anticipated total number of subjects from what was originally approved, or adding any new materials (e.g., letters to subjects, ads, questionnaires).

Unanticipated Problems Involving Risks:  You must promptly report to the IRB any unexpected adverse experience, as defined in the IRB/HRPO policies and procedures, and any other unanticipated problems involving risks to subjects or others.  The Reportable Events Form (REF) should be used for reporting to the IRB.

Audits/Record-Keeping:  Your research records may be audited at any time during or after the implementation of your project.  There are Federal, State and Institutional requirements for record retention.  Check with your organization and your funding agreement to learn more about what record retention requirements apply to your project. 

Additional Information:  Complete information regarding research involving human subjects is available in the “Washington University Institutional Review Board Policies and Procedures.”  Research investigators are expected to comply with these policies and procedures and to be familiar with the Belmont Report, 45CFR46, and other applicable regulations prior to conducting the research.  This document and other important information is available on the HRPO website http://hrpo.wustl.edu/. 


IRB ID #:	202207132

To:		Brian Fuller

Site: 		Washington University in St. Louis

From:		The Washington University in St. Louis Institutional Review Board

Re:	Awareness with Paralysis and Post-Traumatic Stress Disorder among Mechanically Ventilated Emergency Department Survivors: The ED-AWARENESS-2 Trial


Approval Date:	07/05/23
Next IRB Approval
Due Before:	07/03/24

2018 Common Rule/Equivalent Protections Yes

Type of Application:	Type of Application Review:	Approved for Populations:	
 New Project	 Full Board:	 Children	
 Continuing Review	Meeting Date: 	     Signature from one parent	
 Modification	 Expedited	     Signature from two parents	
 Modification to add New Site	 Exempt	 Prisoners	
	 Facilitated	 Pregnant Women, Fetuses, Neonates	
		 Wards of State	
		 Decisionally Impaired	

				

Criteria for approval are met per 45 CFR 46.111 and/or 21 CFR 56.111 as applicable.
Project determined to be minimal risk per 45 CFR 46.102(i) and/or 21 CFR 56.102(i) as applicable.


					   			
Source of Support:	
	NIH, National Heart, Lung and Blood Institute (NHLBI)
		Awareness with Paralysis and Post-Traumatic Stress Disorder among Mechanically Ventilated Emergency Department Survivors: The ED-AWARENESS-2 Trial (Brian M. Fuller, MD, MSCI)
