# Supplementary material for: Awareness with paralysis and symptoms of post-traumatic stress disorder among mechanically ventilated emergency department survivors (ED-AWARENESS-2 Trial): study protocol for a pragmatic, multicenter, stepped wedge cluster randomized trial
Source: Trials. 2023 Nov 25;24:753. doi: 10.1186/s13063-023-07764-5 (PMC10675941; doi:10.1186/s13063-023-07764-5)
Supplement: Supplementary file 2 — Additional file 2. Awareness with Paralysis (AWP) Questionnaire. [file 13063_2023_7764_MOESM2_ESM.docx]

**Awareness with Paralysis (AWP) Questionnaire**

**Assessment of awareness with paralysis (AWP)**

1. We would like to ask you some questions about what you remember while you were on the breathing machine. If you remember, can you tell me about the last thing you remember before you lost consciousness, or before the medical team put you to sleep to put in the breathing tube (in the emergency department, ambulance, or at the scene)?
2. Do you remember anything specifically, such as:

a. Being at home

b. Calling 911

c. The accident or event that caused your illness

d. Hearing voices

e. Being picked up by the ambulance

f. An intravenous (IV) line being placed

g. Feeling a mask on your face

1. What is the first thing you remember when you woke up again?

4. During your medical emergency or anytime you were on the breathing machine, did you ever feel the sensation of being awake but you couldn't move; like you were paralyzed? (Assessment for patients that could have woken up while paralyzed OR remember being paralyzed before losing consciousness, such as memory of intubation.)

- - 1. Tell me about that. What do you remember?
    2. Do you remember the breathing tube being placed in your throat? Describe that please.
    3. Did you try to move? If yes, could you?
    4. Did you try to open your eyes? If yes, could you?
    5. Did you try to breath? If yes, what was your breathing like? (normal, fast, labored, unable to breath)
    6. Did you feel pain like the doctors were doing procedures or operations on you?

1. Pain in your chest or ribs, like doctors were putting a tube in your chest?

2. Pain in your neck, groin, or around collar bone like you were being stuck with a big needle?

3. Pain in your nose, like a tube was being put down the back of your nose?

4. Pain in your throat from the breathing tube?

5. Pain in your genitals, like a small tube (a catheter) was being place where you urinate from?

6. Pain in your arms or legs, like you had a broken bone that was being pulled on?

7. Somewhere else?

1. Where do you think this memory of being paralyzed occurred? (and why)

a. Emergency department

b. Intensive care unit

c. Somewhere else

6. Was this feeling of being paralyzed bad/unpleasant, good/pleasant, or neither (neutral)?

7. If you don’t have a memory of feeling paralyzed, do you have any memory of being on the breathing machine?
