# Supplementary material for: Awareness with paralysis and symptoms of post-traumatic stress disorder among mechanically ventilated emergency department survivors (ED-AWARENESS-2 Trial): study protocol for a pragmatic, multicenter, stepped wedge cluster randomized trial
Source: Trials. 2023 Nov 25;24:753. doi: 10.1186/s13063-023-07764-5 (PMC10675941; doi:10.1186/s13063-023-07764-5)
Supplement: Supplementary file 4 — Additional file 4. World Health Organization Trial Registration Data Set items. [file 13063_2023_7764_MOESM4_ESM.docx]

**Additional File 5**. World Health Organization Trial Registration Data Set items.

| **Data category** | **Information** |
| --- | --- |
| **Primary registry and trial identifying number** | ClinicalTrials.gov (NCT05534243) |
| **Date of registration in primary registry** | 06 September, 2022 |
| **Secondary identifying numbers** | R01 HL162721 |
| **Source(s) of monetary or material support** | National Heart, Lung, and Blood Institute (NHLBI) |
| **Primary sponsor** | National Heart, Lung, and Blood Institute (NHLBI) |
| **Secondary sponsor(s)** | N/A |
| **Contact for public queries** | Brian M. Fuller (fullerb@wustl.edu) |
| **Contact for scientific queries** | Brian M. Fuller (fullerb@wustl.edu) |
| **Public title** | The ED-AWARENESS-2 Trial |
| **Scientific title** | Awareness with paralysis and symptoms of post-traumatic stress disorder among mechanically ventilated emergency department survivors (ED-AWARENESS-2 Trial): study protocol for a pragmatic, multicenter, stepped wedge cluster randomized trial |
| **Countries of recruitment** | United States of America (USA) |
| **Health condition(s) or problem(s) studied** | Awareness with paralysis, post-traumatic stress disorder, depression and anxiety |
| **Intervention(s)** | Control period: usual care.  Intervention period: a multifaceted intervention aimed at reducing the use of rocuronium in the ED and consequently increase the use of succinylcholine; the intervention will be delivered at the cluster level, and the strategies employed revolve around the use of “nudges” without restricting clinician choice. |
| **Key inclusion and exclusion criteria** | Inclusion criteria: 1) mechanical ventilation via an endotracheal tube; 2) age ≥ 18 years; and 3) treatment with a NMB in the ED (for tracheal intubation or in the post-intubation phase of care). Exclusion criteria: 1) acute or chronic neurologic injury with deficit that prevents assessment of AWP (i.e. stroke, intracranial hemorrhage, traumatic brain injury, cardiac arrest, advanced dementia); 2) death before extubation; and 3) transfer to another hospital from the ED. |
| **Study type** | Multi-center, pragmatic, stepped wedge cluster randomized trial  Interventional  Randomized allocation of site order  Purpose: prevention  Phase III |
| **Date of first enrollment** | 28 June, 2023 (actual) |
| **Sample size** | 3,090 (total project enrollment)  199 (currently enrolled as of 24 August, 2023) |
| **Recruitment status** | Recruiting |
| **Primary outcome(s** | Proportion of patients experiencing awareness with paralysis in the control phases versus the intervention phase  Method of measurement: modified Brice questionnaire  Timeframe: assessed after patients are extubated and liberated from the mechanical ventilator |
| **Key secondary outcomes** | Symptoms of post-traumatic stress disorder (PTSD)  Method of measurement: PCL-5  Timeframe: 30 and 180 days after hospital discharge  Symptoms of depression and anxiety  Method of measurement: HADS  Timeframe: 30 and 180 days after hospital discharge |
| **Ethics review** | Status: approved  Date of approval: 17 August, 2022  Washington University in St. Louis Human Research Protection Office (email: hrpo@wustl.edu/) |
| **Completion date** | Pending |
| **Summary results** | Pending |
| **IPD sharing statement** | We will follow current data sharing policies of the funding agency in order to share data with other investigators through academically established means. |
